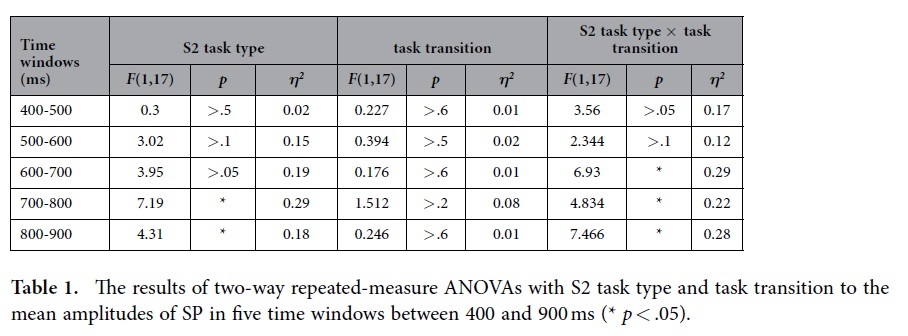# The neural dynamic mechanisms of asymmetric switch costs in a combined Stroop-task-switching paradigm

**DOI:** 10.1038/srep10240

**Published:** 2015-05-20

**Authors:** Shanshan Wu, Glenn Hitchman, Jinfeng Tan, Yuanfang Zhao, Dandan Tang, Lijun Wang, Antao Chen

**Affiliations:** 1 Key Laboratory of Cognition and Personality of Ministry of Education, Faculty of Psychology, Southwest University, Chongqing 400715, China

## Abstract

Switch costs have been constantly found asymmetrical when switching between two tasks of unequal dominance. We used a combined Stroop-task-switching paradigm and recorded electroencephalographic (EEG) signals to explore the neural mechanism underlying the phenomenon of asymmetrical switch costs. The results revealed that a fronto-central N2 component demonstrated greater negativity in word switch (cW) trials relative to word repeat (wW) trials, and both First P3 and P3b components over the parieto-central region exhibited greater positivity in color switch (wC) trials relative to color repeat (cC) trials, whereas a contrasting switch-related fronto-central SP effect was found to have an opposite pattern for each task. Moreover, the time-frequency analysis showed a right-frontal lower alpha band (9-11 Hz) modulation in the word task, whereas a fronto-central upper alpha band (11-13 Hz) modulation was exclusively found in the color task. These results provide evidence for dissociable neural processes, which are related to inhibitory control and endogenous control, contributing to the generation of asymmetrical switch costs.

Human beings have the ability to alternate flexibly between different goals and actions, in response to ever-changing contextual demands. In task-switching paradigms, response times (RTs) are consistently found to be longer on task switch trials as compared with task repeat trials, and this deficit is typically referred to as the switch cost^1^,^2^. Notably, the cost is often asymmetrical when shifting between two tasks with different levels of dominance. As an example, in a color-word Stroop task^3^, where subjects are instructed to read a color word ("the dominant task") or name the ink color ("the nondominant task"), greater switch costs are observed when switching from color naming to word reading than vice-versa^4^. This paradoxical finding is termed asymmetric switch costs, which has been replicated across a variety of studies^5^,^6^,^7^,^8^.

Allport and colleagues^4^,^9^ have put forward a hypothesis that attempts to account for asymmetries in switch costs. They suggest that the switch cost reflects the time needed to resolve interference resulting from a persisting activation of a previously active task-set in task switching (i.e., *task-set inertia*). The task-set refers to some specific cognitive operations that enable the person to perform a task effectively. Thus, the task-set must include the representation of stimuli, responses and the corresponding stimulus-response (S-R) mappings relevant to the executed tasks^10^. According to the task-set inertia account, the asymmetric switch costs can be attributed to the different carryover effects of the performance of previous tasks on subsequent trials. For instance, when subjects are instructed to name the color of an incongruent Stroop stimulus, the inhibition of competing word processing functions may be required because the word reading task is more dominant. This inhibition will persist into the next trials and disturb the execution of the subsequent task in which word reading is the now-relevant task, leading to large switch costs for word reading. However, there will be little or no inhibition of color processing during word reading performance, so that no persisting inhibition needs to be overcome, resulting in small switch costs for color naming.

The task-set inertia account emphasizes the difference in the requirements for overcoming the persisting task-set inhibition. However, some other researchers suspect the role of inhibition in asymmetric switch costs. For example, Yeung and Monsell^11^ developed a computational model, assuming the asymmetric switch costs reflect a combination of the task priming effect and top-down control input. Task priming is analogous to the concept of task-set inertia, which represents a carryover effect from prior task-sets. Top-down control input serves to give top-down activation of the relevant task-set to ensure appropriate task performance. In this model, the control inputs for the non-dominant task are greater than the dominant task because the former is assumed to have lower baseline activation. The performance of the dominant task switch is prolonged by negative task priming from prior non-dominant tasks, relative to the dominant task repetition. In contrast, the negative task priming is attenuated by high control inputs when switching to the non-dominant task, reducing the switch costs for the non-dominant task. Therefore, asymmetric switch costs primarily result from the difference in the strength of top-down control input between the dominant task and the non-dominant task.

There is persuasive neural evidence in support of each proposal. An event-related potential study showed a late frontal negativity (LFN) was present during preparation for a switch to the dominant task, but was absent when switching to the non-dominant task^12^. The authors propose that this effect reflects the differential persistence of task-set inhibition. In a language switching study, a switch-related N2 component over the frontal cortices showed an asymmetry across first and second languages. This effect was interpreted to reflect the suppression of the more habitual response^13^. However, some other switch-induced late components (e.g., P3) were observed in prior studies, which have been regarded as a possible neural signature of top-down control for establishing a new task-set in task switching^14^,^15^,^16^.

As can be seen from the above findings, the asymmetric switch cost may not be a purely inhibitory phenomenon^5^. Some researchers have therefore posited hybrid accounts which suggest that asymmetric switch costs may reflect both inhibitory processes and top-down control^17^,^18^. Based on this suggestion and the former studies, we speculated that both inhibitory control and endogenous control would be involved in switching between a pair of tasks with unequal dominance, and that inhibitory control would task place before endogenous control. Specifically, in the context of task switching, the inhibitory control is conceptualized as the ability to overcome the persisting task-set inhibition, and the endogenous control refers to the processing of establishing a new task-set, including resolving interference from previous task-set and top-down control to activate the currently relevant task-set. In order to test this assumption, we combined the color-word Stroop task with a pair-wise task sequence paradigm. There are two reasons for this. First, due to the intensive study of the Stroop effect, the nature of the Stroop color naming/word reading tasks is well understood. Thus, more definitive conclusions can be drawn by studies employing these tasks. A second reason is that studies concerning the effects of switching between color naming and word reading are relatively ample^4^,^19^,^20^, making it easy to compare the results from our experiment with previous data. Unlike the widely used task-cuing paradigm, in which a task cue is presented before the target appears, the pair-wise task sequence paradigm we adopted presents the cue and target simultaneously, and each trial contains a pair of successive tasks that are either the same or different. In this case, the task sequence is unpredictable. Meanwhile, there is no difference in task preparation because it is not possible to prepare for switch or repeat trials^21^. Hence, the switch costs we obtained would not be confounded by the effects of anticipatory preparation processes.

Accurate changes in brain activation can be recorded through high temporal resolution ERPs. However, the electrophysiological signals have also been investigated by characterizing frequency-specific oscillatory activity induced by neuronal synchronization. In general, the modulations of oscillatory activity involve either a decrease (event-related desynchronisation, ERD) or increase (event-related synchronization, ERS) in a certain frequency band. It is widely accepted that ERS and ERD are associated with cortical activation, inhibition, and binding processing^22^. Particularly, the ERD in the alpha band (8-13 Hz) has been shown to play an important role in task switching^23^,^24^,^25^. Therefore, we could examine the role of neural oscillations in switching between the color task and the word task.

The goal of the present study was to investigate the temporal dynamics of processes contributing to asymmetric switch costs by analyzing neurophysiological parameters derived from ERPs and complement this approach with time-frequency analysis. The modulation of oscillatory activity was represented by event-related spectral perturbations (ERSPs) and was estimated using a continuous wavelet transform (CWT). Importantly, our analyses focused on the differences in neural activity during the presentation of the second stimulus in a task pair between switch and repeat trials; of interest was whether switch-related neural activity would differ across the color task and the word task. We predicted there would be a switch-related N2 effect confined to the word task reflecting the overcoming of the persisting task-set inhibition, and this effect was not expected to be evident in the color task. Moreover, we expect that the engagement of endogenous control would be reflected by a parieto-central P3b component and a sustained potential (SP). The P3b is a subcomponent of the P300 and is believed to be related to context updating^26^. The SP is a lateral frontal negativity or a sustained parietal positivity related to the resolution of Stroop-like conflict or the implementation of attentional control^27^,^28^,^29^. According to our hypothesis, endogenous control is preceded by inhibitory control. We expected that the N2 component to be followed by the P3b and the SP components. On the basis of previous time-frequency studies^23^,^24^, we expected a modulation of alpha activity would be obtained during task switching.

## Results

### Behavioral data

**Figure 1:**
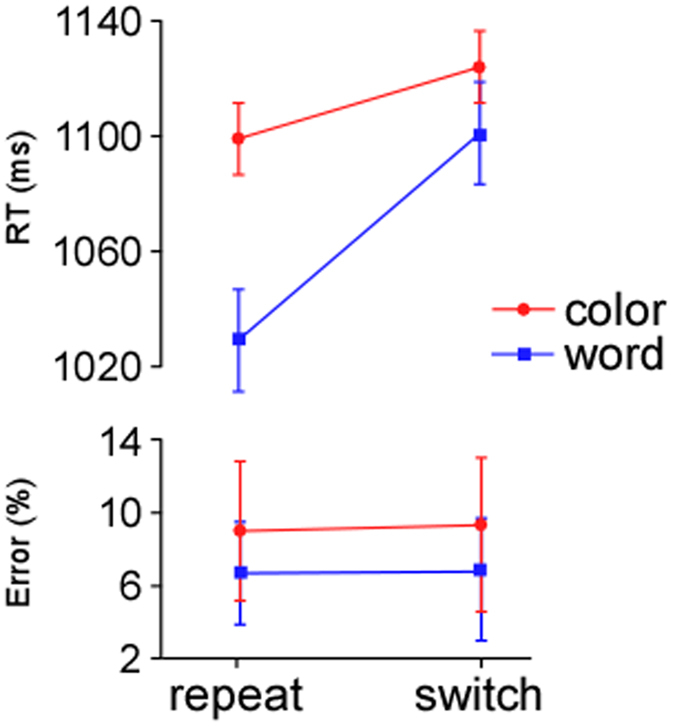
The mean RTs (top) and error rates (bottom) for the color task and the word task as a function of task sequence.

We found a marginally significant main effect of S2 task type (*F* (1, 17) = 4.09, *p* < .06, *η*^*2*^ = .19), showing that responses for the word task (mean, 1065 ms; SD, 40) were faster than the color task (mean, 1112 ms; SD, 37). The factor of task transition was also significant (*F* (1, 17) = 19.24, *p* < .0001, *η*^*2*^ = .53), with responses on switch trials (mean, 1113 ms; SD, 38) being slower than on repeat trials (mean, 1064 ms; SD, 36). Importantly, there was a reliable interaction between S2 task type and task transition (*F* (1, 17) = 4.63, *p* < .05, *η*^*2*^ = .21). *Post-hoc* tests showed that the switch costs for the word task (mean, 71 ms; SD, 18) were larger than for the color task (mean, 26 ms; SD, 12) (*F* (1, 17) = 4.51, *p* < .05; see Fig. 1, top), replicating the asymmetry observed in previous studies^4^. Moreover, the RTs were faster in the wW trials than in the cC trials (*F* (1, 17) = 7.64, *p* < .05, *η*^*2*^ = .31), but no significant difference between the cW and wC trials was observed. For the error rates, the main effect of S2 task type was significant (*F* (1, 17) = 8.48, *p* < .01, *η*^*2*^ = .33): participants produced more errors when performing the color task (mean, 5.3%; SD, 3.9%) than the word task (mean, 3.2%; SD, 3.1%) (see Fig. 1, bottom). No other main effects or interactions were significant (all *Fs* < 2.8, *ps* > .1).

### ERP data

**Figure 2:**
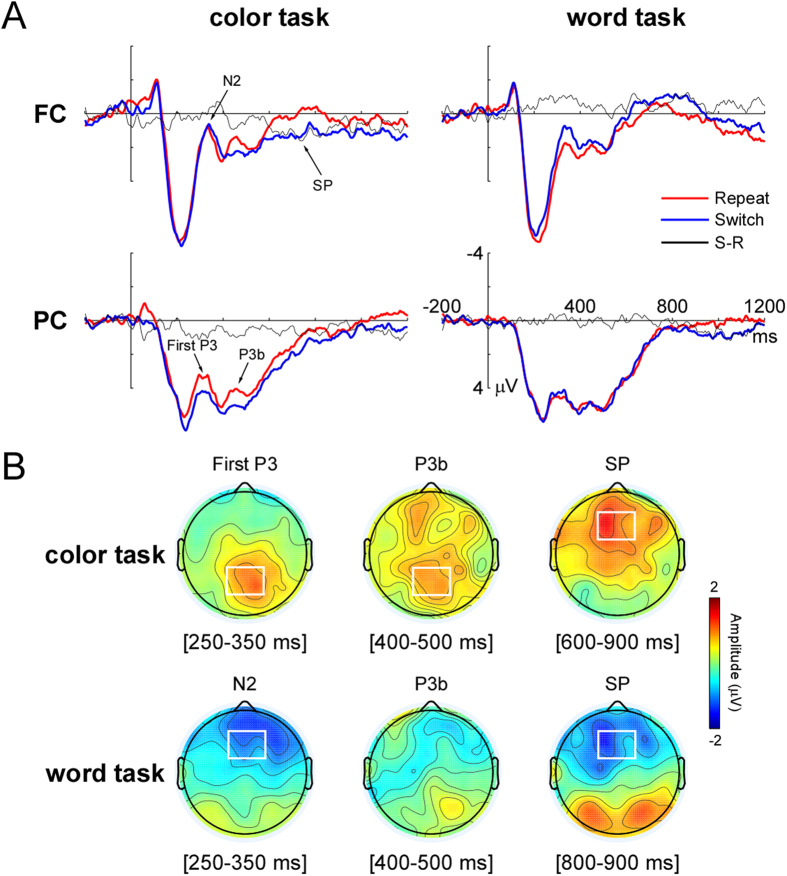
The ERP results. **A:** The grand-averaged S2-locked ERP waveforms elicited by switch and repeat trials for the color task (left) and for the word task (right) at fronto-central sites (FC, top) and at parieto-central sites (PC, bottom), respectively. **B:** Topographic maps show voltage differences between switch and repeat trials (switch minus repeat) for all ERP components in both tasks. The significant switch-related ROIs are outlined in the rectangles.

Figure 2 illustrated the grand-averaged ERPs waveforms at select electrodes and the scalp topographies of difference wave (switch minus repeat) for the N2, First P3, P3b and SP.

For the P2 component, the S2 task type × task transition interaction was significant (*F* (1, 17) = 5.88, *p* < .05, *η*^*2*^ = .24). *Post-hoc* tests showed that there was significant difference between wC and cW trials (*F* (1, 17) = 5.44, *p* < .05, *η*^*2*^ = .22). No main effects achieved significance (S2 task type: *F* (1, 17) = .08, *p* > .7; task transition: *F* (1, 17) = .17, *p* > .6).

The analysis of the N2 revealed that there was a significant S2 task type × task transition interaction (*F* (1, 17) = 6.67, *p* < .05, *η*^*2*^ = .28) in the 250-350 ms time window. This interaction resulted from a significant difference in the amplitude of the N2 between wW and cW trials (*F* (1, 17) = 14.69, *p* < .001, *η*^*2*^ = .46), and no difference between cC and wC trials (*F* (1, 17) = .53, *p* > .4). Thus, the switch-specific modulation of N2 was observed only for the word task.

Concerning the First P3 component in the 250-350 ms time window at the parieto-central region, a significant S2 task type by task transition interaction (*F* (1, 17) = 8.20, *p* < .05, *η*^*2*^ = .33) revealed the presence of task transition effect in the color task (*F* (1, 17) = 7.37, *p* < .05, *η*^*2*^ = .30), with more positive amplitude in wC than in cC trials, but not in the word task (*F* (1, 17) = 3.48, *p* > .07). For the analysis of P3b component in the 400-500 ms time window, a significant S2 task type by task transition interaction (*F* (1, 17) = 6.55, *p* < .05, *η*^*2*^ = .28) revealed that P3b amplitudes were enhanced in wC trials compared with cC trials (*F* (1, 17) = 9.04, *p* < .01, *η*^*2*^ = .35). However, there was no difference between the cW and wW trials (*F* (1, 17) = .85, *p* > .3). These results indicated that the switch-related modulations of First P3 and P3b were only evident for the color task.

For SP analyses, Window (5 modalities) was added into the two-way ANOVAs. The results revealed a significant main effect of the S2 task type (*F* (1, 17) = 5.15, *p* < .05, *η*^*2*^ = .23), suggesting the difference in the amplitude of SP between the color task and word task was significant in five time windows between 400 and 900 ms after S2 onset. There was also a marginally significant interaction among Window, S2 task type and task transition (*F* 4, 14) = 2.38, *p* = .06, *η*^*2*^ = .12). Follow-up ANOVAs showed the interaction between S2 task type and task transition was significant in three time windows between 600 and 900 ms (all *Fs* >4.8, *ps* <.05, *η*^*2*^*s* >.2, see Table 1 for detailed results in all five time windows), but not in two time windows between 400 and 600 ms (400-500 ms: *F* (1, 17) = 3.56, *p* > .07; 500-600 ms: *F* (1, 17) = 2.34, *p* > .1). *Post-hoc* tests uncovered SP amplitudes were larger in wC trials than in cC trials at three time windows in a 600-900 ms time range (all *Fs* > 6, *ps* < .05, *η*^*2*^*s* >.2), however, the SP was more negative in cW trials than in wW trials, and this effect was only significant at the 800-900 ms time window (*F* (1, 17) = 5.42, *p* < .05, *η*^*2*^ = .22).

### Time-frequency data

**Figure 3:**
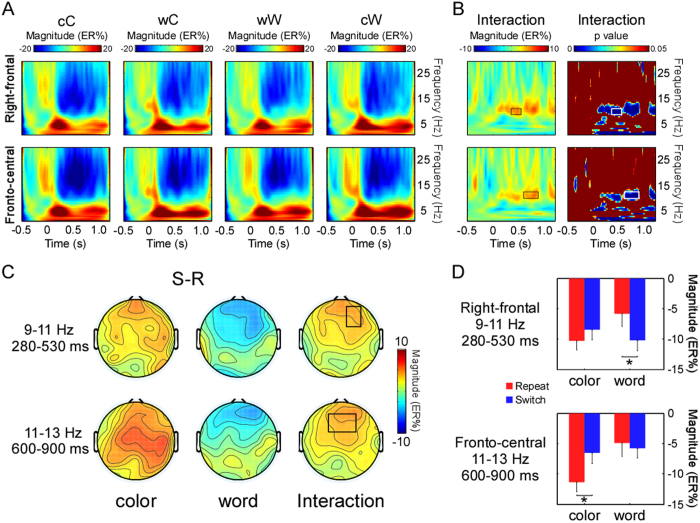
The time-frequency results. **A:** The grand-average time-frequency representations (expressed as ER %) for each condition (cC, wC, wW, cW) in the right-frontal sites and fronto-central sites, respectively. **B:** The grand-average time-frequency representations of the interaction between S2 task type and task transition condition and the bootstrapping statistical analysis at the significance level of *p* < .01 (FDR corrected) based on the interaction time-frequency representations in the two S-ROIs. The defined TF-ROIs are outlined in the rectangles. The lower alpha band (9-11 Hz) was defined in the right-frontal (280-530 ms) sites and the upper alpha band (11-13 Hz, 600-900 ms) was defined in the fronto-central sites. **C:** The difference scalp topographies of ERSP magnitudes between switch and repeat trials (S-R) in both tasks and scalp topographies of ERSP magnitudes of interaction within the defined TF-ROIs. **D:** The mean ERSP magnitudes of the lower and upper alpha bands within the defined S-ROIs for both tasks (* *p* < .05).

The grand-average time-frequency representations in the two S-ROIs are illustrated in Fig. 3A. The TF-ROIs in the lower alpha band (9-11 Hz, 280-530 ms) and upper alpha band (11-13 Hz, 600-900 ms) that displayed the most pronounced interaction effects were defined (shown as rectangles in Fig. 3B, *p* < .05, FDR corrected). The difference scalp topographies of ERSP magnitudes for cC, wC, wW and cW trials in the two TF-ROIs are illustrated in Fig. 3C.

The modulation of the lower alpha band (9-11 Hz) mainly occurred in the right- frontal region within the interval of 280-530 ms post-S2. The results of the two-way ANOVA showed a significant interaction between S2 task type and task transition (*F* (1, 17) = 8.09, *p* < .05, *η*^*2*^ = .32). *Post-hoc* tests revealed that the lower alpha band ERD was significantly stronger in the cW than in the wW trials (*F* (1, 17) = 7.39, *p* < .05, *η*^*2*^ = .3). However, the mean magnitudes of the lower alpha band ERD between the wC trials and cC trials were not significant (*F* (1, 17) = 3.53, *p* > .05).

The modulation of the upper alpha band (11-13 Hz) mainly occurred in the fronto-central regions during the 600-900 ms period after S2 onset. The ANOVAs revealed a main effect of S2 task type (*F* (1, 17) = 4.88, *p* < .05, *η*^*2*^ = .22) and a significant interaction between the S2 task type and task transition (*F* (1, 17) = 9.14, *p* < .01, *η*^*2*^ = .35). *Post-hoc* tests confirmed that the upper alpha band ERD was significantly stronger in the cC than in the wC trials (*F* (1, 17) = 10.24, *p* < .01, *η*^*2*^ = .38), but was not statistically significant between the wW and cW trials (*F* (1, 17) = 1.47, *p* > .2).

## Discussion

Employing a pair-wise task sequence paradigm that incorporated modified Stroop stimuli and using multi-methodological approach that combined ERPs with time frequency analysis, we examine the neurophysiological mechanisms underlying switching between two tasks of unequal dominance. In congruence with previous findings^4^,^11^,^20^, the RT pattern of asymmetrical switch costs observed in the current study manifested as larger switch costs for the word task than for the color task. Our ERP and time-frequency results showed that a switch-related N2 effect and a lower alpha power (9-11 Hz) over the right-frontal scalp sites were only apparent for the word task. These findings indicate that an inhibitory control mechanism was involved in overcoming the persisting task-set inhibition during switching from the color task to the word task, which is consistent with the task-set inertia account. Additionally, we found a First P3 and a P3b component at the parieto-central regions, an SP component and an upper alpha power (11-13 Hz) at the fronto-central regions which confined to the color task. These findings may suggest that stronger endogenous control needs to be implemented during switching to the color task so that the newly relevant task-set can be established.

As expected, a more negative deflection of N2 component over the fronto-central region in the cW trials relative to the wW trials was observed. We propose that this result may reflect the process of overcoming persisting task-set inhibition which was applied during the previous color task performance. The conflict would have been encountered in each trial because all stimuli in the present experiment were incongruent. Thus, it was necessary for participants to suppress the strong tendency to identify the word when performing the color task, and this inhibition was able to persist into subsequent trials and needed to be overcome when the word task-set was appropriate, producing a cost of switching to the word task. By contrast, there was no need to suppress the color information when performing the word task and no inhibition was needed to be overcome during switching to the color task, so the switch-related N2 effect was not present in the color task. This N2 component resembles the LFN which was reported in a study using a prosaccade/antisaccade switching task^12^. Because the prosaccade task has been generally considered to be more dominant than the antisaccade task, the authors propose that the switch-specific LFN confined to the presaccade task reflects the recovering or overcoming of persisting inhibition, which is in concordance with our assumption. It is noteworthy that performing both repeat and switch trials in the current study may result in stimulus and response conflict, potentially leading to stimulus and response suppression in both the task repetition and task switching conditions. Therefore, the present N2 modulation might not be attributed to the response suppression^13^,^30^ or the size of response conflict^31^,^32^.

The time-frequency analysis indicated an increased lower alpha band (9-11 Hz) ERD in the cW trials relative to the wW trials. A previous study found a lower alpha band ERD after a warning signal and before the onset of the imperative stimulus, suggesting that the lower alpha band ERD is critically related to attentional demands such as alertness^33^. Following this interpretation, our result may indicate that performers raised their level of alertness during switching to the word task, reflecting the requirement of attentional resources to overcome persisting inhibition.

We also observed the amplitudes of the parieto-central First P3 and P3b component were larger in the wC trials than in the cC trials, and these effects were not evident in the word task. The First P3 component has been observed in a study of the timing of the color-word semantic conflict involved in the Stroop effect^34^ and has been thought to belong to the P300 “family”. According to Zurrón and colleagues^34^, the First P3 effect in response to incongruent stimuli may indicate the temporal locus of the semantic conflict between the meaning of the word and the meaning of the color. Taking into account that persisting activation of the previously activated task-set will interfere with the execution of the current performance in the context of task switching^4^,^9^, the switch-related First P3 modulation in the interval between 250 and 350 ms after S2 onset may reflect the temporal locus of the interference between previous task-set and current task-set. Because the word task was more dominant than the color task, the activation of the color task-set would be more interfered by the persisting activation of the previous word task-set, whereas the activation of the word task-set would be less interfered by the previous color task-set. The differing levels of task-set interference could be responsible for the absence of First P3 modulation in the word task.

Most ERP studies using task-cuing paradigms reported that an increased positivity in P3b amplitudes in switch trials after cue onset may reflect the anticipatory task-set reconfiguration process^14^ or updating of the context in working memory during task switching^35^, while less pronounced P3b amplitudes found in switch trials after target onset may reflect a greater amount of cognitive resources required in task switching^36^. Based on the above assumption on the First P3 modulation, we consider that the target-locked P3b effect observed in the color task may reflect the resolution of the interference from previous word task-set. The latency difference between First P3 and P3b may reflect minimal time requirement between task-set interference detection and resolution. Theoretically, the resolving of interference from a previous task-set is described as an aspect of the endogenous control processes establishing a task-set within the context of switching. Thus, we can assume that the P3b effect is likely a neural marker of the initiation of endogenous control during the color task switch. Meanwhile, the absence of the P3b effect for the word task may reflect less endogenous control required for interference resolution during the word task switch.

The modulation of SP over the fronto-central region started at 600 ms after S2 onset for the color task, whereas it started at 800 ms after S2 onset for the word task. More specifically, the SP amplitudes elicited by the cW trials were more negative-going than the wW trials; however, the pattern of the SP effect for the color task was the opposite (the SP amplitudes were more negative-going in the cC trials than in the wC trials). The SP is a sustained negativity over the lateral frontal regions or sustained positivity over the centro-parietal region^27^,^28^,^29^,^37^. Previous studies have indicated that the modulation of SP over the centro-parietal was associated with the controlled processing of Stroop-like conflict resolution^27^,^29^ and conflict adaptation^38^. However, there is still a lack of studies concerning the frontal SP and there has been little evidence to show that this component is relevant to the processing of task switching. As we hypothesized, the modulation of SP observed in the current study may suggest that endogenous control is required to activate the currently relevant task-set before response execution, which is thought to belong to another part of task-set establishment^39^,^40^. Given that the difference in the SP amplitudes between switch and repeat trial was greater for the color task than for the word task, it may be easier to establish the dominant word task-set than the non-dominant color task-set. However, compared to the color task, the pattern of the SP effect for the word task was reversed. This effect seems to indicate that the process involved in overcoming the persisting task-set inhibition postpones the establishment of the new task-set, and it has a continuing impact on the activation of the word task-set, producing a large switch cost for the word task in behavior. Remarkably, the present study found that the modulation of SP was most pronounced over the fronto-central region, inconsistent with some previous results indicated that the topography distribution of conflict SP mainly implicated the centro-parietal region. We propose that the fronto-central SP may reflect the processing of task-set activation. Therefore, the modulation of fronto-central SP observed in our study may provide informative evidence for the role of SP in task switching research.

Concerning the time-frequency analysis, we found that the magnitudes of upper alpha band (11-13 Hz) ERDs were higher in the cC trials compared with the wC trials in the fronto-central region. It has previously been suggested that the decrease in upper alpha band power may be correlated with search and retrieval processes in long-term memory^41^ which has been regarded as a kind of cognitive operation during task switching^2^. If this is indeed the case, the upper alpha band ERDs should be higher in the wC trials, which was not observed in the current study. However, our results also revealed an increased upper alpha band power in the wC trials relative to the cC trials. We propose that endogenous control may be implemented to activate the color task-set in the wC condition, which fits well with a previous assumption that the increase in the alpha frequency power is closely related to the top-down control of cortical activation^42^. Furthermore, there was no significant difference between the power of upper alpha band in wW and cW trials. This result may suggest that the activation of the word task-set calls for less endogenous control.

In sum, the current findings provide direct neural evidence for two temporally dissociable processes, inhibitory control and endogenous control, underlying switching between two tasks with unequal dominance. Inhibitory control is engaged in overcoming the persisting task-set inhibition and endogenous control is engaged in establishing the new task-set. Given the timing of the neural activities and their sensitivity to each process, the inhibitory control appears to takes place before the endogenous control. Integrating our behavioral and electrophysiological results, we suggest that inhibitory control is a more time-consuming process which only takes place during switching to the word task, so the switch costs for the word task obtained in behavioral results are robust. By contrast, the recruitment of endogenous control processes improves performance when switching from the word task to the color task, resulting in relatively small switch costs for the color task. Therefore, we conclude that asymmetric switch costs can be caused by a combination of factors related to inhibitory control and endogenous control. Our findings shed light on the relative contributions of these functions to the generation of asymmetric switch costs and may push forward the development of existing theory.

## Methods

### Participants

Eighteen students without any psychiatric, neurological, or medical illness from Southwest University (8 female, 21.3 ± 1.3 years old) took part in this study for monetary compensation. All participants were right-handed, had normal or corrected-to-normal vision, without achromatopsia or color weakness. The study was approved by the Human Research Ethics Committee of the Southwest University of China and was in accordance with the ethical guidelines of the Declaration of Helsinki. All signed informed consent before the experiment. In addition, the participants were blind to the purpose of the experiment.

### Stimuli and procedure

The stimuli consisted of color words (RED, YELLOW, GREEN, and BLUE, in Chinese presented in the Song Ti font) written in black superimposed on a background shape which was filled with one of the four colors (red, yellow, green, blue). Only incongruent stimuli were used, where the color of the background did not coincide with the word meaning (e.g., the word RED displayed on a green background). The participant’s task was to respond to either the color of the background or the name of the word, which was instructed by alternating the shape of the background (visual angle; 1.5° × 1.5°) between a square and a diamond (i.e., a square rotated by 45°). The square indicated the color task (i.e., respond according to the color of the background and regardless of the name of the word), whereas the diamond indicated the word task (i.e., respond according to the name of the word and regardless of the color of the background). The associations between task type and background shape were counterbalanced across participants.

Participants were required to respond as quickly and accurately as possible, by pressing one of four keys (D, F, J, or K, separately corresponding to one of the four colors/words) on a computer keyboard with their index and middle fingers of their right and left hands (the finger assigned to each specific target was counterbalanced across participants). Before launching the practice, an initial selection for participants was conducted to ensure the fully understanding of the instructions. Participants needed to perform a color/word mapping task with 64 trials of a colored X or a color word written in black (four colors and words above mentioned). Participants responded to the color of the X or the name of the word by pressing the corresponding key. Only when their correct rates are greater than 90%, can the participants take part in the practice and experiment.

**Figure 4:**
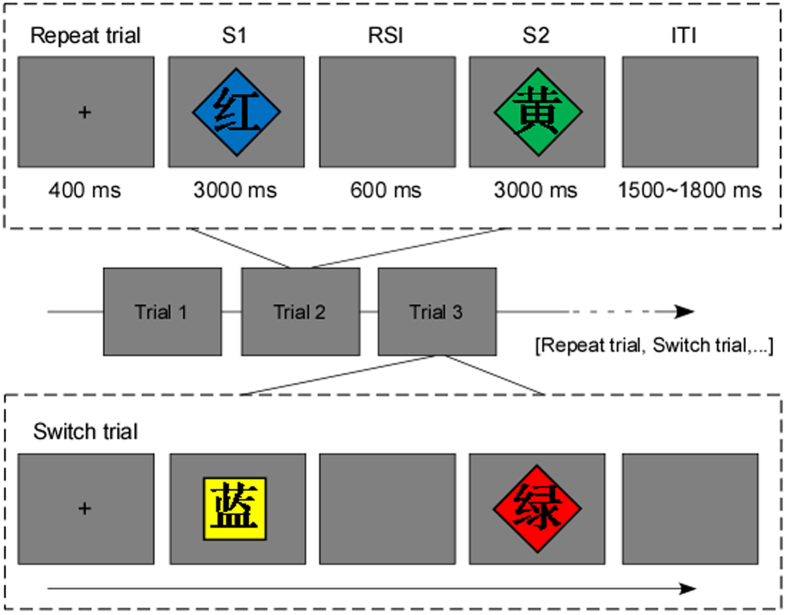
Illustration of the stimulus materials and the order of a repeat trial and a switch trial (RSI: response-stimulus interval; ITI: intertrial interval).

Being preceded by 16 practice trials, the experiment consisted of 320 trials separated into four blocks. Each trial comprised of two task stimuli. On a repeat trial, the background shape of the first stimulus (S1) was the same as the second stimulus (S2), indicating the task was identical in S1 and S2. On a switch trial, the background shape of S1 was different from that of S2, indicating the task was different in S1 and S2. To assess asymmetric switch costs, trials were subdivided into four types: color task repetition (cC), color task switching (wC), word task repetition (wW), and word task switching (cW), with each type being randomly presented with equal frequency. The order of a trial was as follows (see Fig. 4): a black cross serving as a fixation appeared at the center of the screen for 400 ms, then the S1 was presented for 3000 ms (or until a response was recorded). After a 600 ms response-stimulus interval (RSI), the S2 was presented for 3000 ms (or until a response was recorded), and the interval between two successive trials (ITI) varied randomly between 1500 and 1800 ms. To avoid confounding response switching with task switching, the correct responses in reaction to the two successive stimuli on each trial were always different, even on task repeat trials.

### Electroencephalographic recordings

Participants were tested in a dimly illuminated and sound-attenuated room. EEG activity was continuously recorded from 64 scalp sites using tin electrodes mounted on an elastic cap based on the extended 10/20 system (Brain Products, GmbH, Germany; pass band: 0.01-100 Hz, sampling rate: 500 Hz). All recording sites were referenced to a channel located on FCz and also re-referenced offline to the average of the left and right mastoids. The horizontal and vertical Electro-Oculogram (EOG) signals were recorded from two additional electrodes, which were placed on the outer canthus of and below the right eye. All impedances were maintained below 5 kΩ. Data were filtered offline with a pass of 0.01-30 Hz for offline analysis. Trials with EOG artifacts (ocular movements and eye blinks), artifacts because of amplifier clippings, bursts of electromyography activity, or peak-to-peak deflections exceeding ±80 μV were rejected offline.

### Analyses of behavioral data

The first trial of each block, trials with incorrect responses (note: only trials with correct responses to both S1 and S2 were considered correct), and trials associated with too fast (<100 ms) and too slow (>2.5 SDs above participants’ mean RT) responses were excluded from further statistical analyses. These data-trimming procedures resulted in the exclusion of 3.1% of the total trials. All post-hoc tests of simple effects were performed using the Bonferroni correction with a significance level of *p* < .05.

### Analyses of ERP data

The Vision Analyzer software package (Brain Products GmbH) was used for the ERP analyses. Because of the present purpose, we only analyzed the ERP epochs which were locked to the presentation of S2, with each epoch included 200 ms pre-S2 (baseline corrected) to 1200 ms post-S2 activity. After removing trials with EEG artifact and data trimming, the mean number of trials used to compute average ERP waveforms ranged across task transition from 65.6 (SD = 8.2) to 65.8 (SD = 8.8) for the color task, and from 61.8 (SD = 8.4) to 63.1 (SD = 7.9) for the word task. Based on the previous task switching and Stroop studies^34^,^43^,^44^ and visual inspection of the scalp distribution maps of the present data, the following ERP components, time windows and scalp region-of-interests (ROIs) were selected for statistical analyses. Mean amplitudes were measured within a time window of 180-220 ms after S2 onset for P2 and 250-350 ms for N2 at fronto-central sites (Fz, F1, F2, FC1, FCz, and FC2). Similar analysis time window and electrode location were used in West and colleagues^44^. The amplitudes of the First P3 component were measured within a time window of 250-350 ms post-S2 at parieto-central sites (CPz, CP1, CP2, Pz, P1, and P2). The amplitudes of the P3b component were measured as the mean voltage in the windows of 400-500 ms post-S2 at the same electrodes as the First P3. The mean amplitudes of SP were measured using fronto-central sites (Fz, F1, F2, FC1, FCz, and FC2). Because the SP component was a long wave with a 500 ms time course, mean amplitudes were also calculated at five 100 ms-lasting time windows for this component (from 400 to 900 ms). Statistical analyses were carried out by using repeated measures analyses of variance (ANOVAs) with S2 task type (the color task, the word task) and task transition (repeat, switch) as within-subjects factors. Because of the five time windows in the analyses of SP components, we added Window as a factor in the above ANOVAs. The Greenhouse-Geisser correction was applied when appropriate. A Bonferroni-corrected significance level of p < .05 was adopted for examination of simple effects.

### Analyses of time-frequency data

EEG data were preprocessed using the EEGLAB toolbox^45^. Epochs comprised a 1700 ms time window, including 500 ms prior to S2 onset and baseline correction was performed using the pre-S2 time interval. Oscillatory power, estimated as a function of time and frequency (time-frequency representation), was obtained from single trial EEG epochs using the CWT conducted by Letswave software ( http://www.nocions.org/letswave/)^46^. The parameters of central frequency (ω) and restriction (σ) in CWT were 5 and 0.15, respectively, and time-frequency representations were explored between 1 Hz and 30 Hz in steps of 0.58 Hz. Single trial time-frequency representations were then averaged to obtain averaged time-frequency representations, which were used to identify the modulations of ongoing EEG rhythms (ERSP). For each estimated frequency, ERSP magnitudes were displayed as an increase or decrease in oscillatory power relative to the pre-S2 interval (-0.45 s to -0.05 s) according to the following formula: ER_*t, f*_ % = [A_*t, f*_ -R_*f*_]/R_*f*_, where A_*t, f*_ is the signal power at a given time (*t*) and frequency (*f*), and R_*f*_ is the signal power averaged within the pre-S2 interval^22^. To avoid edge effects when performing CWT, the pre-S2 time interval (-0.45 s to -0.05 s) was used as a baseline interval. The grand-average time-frequency representations were computed for the cC, wC, wW, cW trials at the single subject level.

Because the most pronounced interactions were observed in the right-frontal sites (AF4, F2 and F4) and fronto-central sites (Fz, F1, F2, FCz, FC1 and FC2), we selected these regions as the spatial regions of interest (S-ROIs) for subsequent quantitative analysis. The time-frequency regions of interest (TF-ROIs) were defined based on a data-driven exploratory analysis strategy, as follows:

1. Based on the defined S-ROIs, we calculated the difference in the ERSP magnitude according to the formula [(cW-wW)-(wC-cC)] (expressed as ER % in the three S-ROIs) to evaluate ASCs.
2. For each obtained time-frequency representation of the ERSP magnitude difference (for the interaction between S2 task type and task transition), we tested whether and when the resulting ERSP magnitudes in the post-S2 interval were significantly different from the ERSP magnitudes in the pre-S2 interval using a bootstrapping method^45^,^47^. At each time-frequency point in the post-S2 interval, investigated populations and reference populations were collected from eighteen participants. The null hypothesis was that there was no mean difference between the two populations. The pseudo-t statistic between the two populations was calculated, and we estimated the probability distribution of the pseudo-t statistic by sampling with two replacement populations of the same size from the reference population. The permutation was executed 5000 times. The distributions of the pseudo-t statistics from the reference population and the bootstrap *p* value for the null hypothesis were generated.
3. This procedure revealed time-frequency distributions in which the brain responses within the post-S2 interval were significantly different from the responses in the reference interval^48^. To address the problem of multiple comparisons, the significance level (*p* value) was corrected using a false discovery rate (FDR) procedure^47^. In addition, to control for false-positive observations^49^, significant TF-ROIs were defined based on the following two criteria: (a) the time-frequency pixels were significantly different from the pre-S2 interval at *p* < .05; and (b) the time-frequency pixels had to include more than 125 consecutive significant time points (0.25 s)^50^,^51^.

The mean magnitude within the identified TF-ROIs at corresponding S-ROIs for each experimental condition was calculated and delivered to a two-way repeated-measures ANOVAs with factors “S2 task type” and “task transition”.

## Additional Information

**How to cite this article**: Wu, S. *et al.* The neural dynamic mechanisms of asymmetric switch costs in a combined Stroop-task-switching paradigm. *Sci. Rep.* **5**, 10240; doi: 10.1038/srep10240 (2015).

## Figures and Tables

**Table 1:**